# *E. coli* Surface Properties Differ between Stream Water and Sediment Environments

**DOI:** 10.3389/fmicb.2016.01732

**Published:** 2016-11-01

**Authors:** Xiao Liang, Chunyu Liao, Michael L. Thompson, Michelle L. Soupir, Laura R. Jarboe, Philip M. Dixon

**Affiliations:** ^1^Department of Agricultural and Biosystems Engineering, Iowa State University, AmesIA, USA; ^2^Department of Microbiology, Iowa State University, AmesIA, USA; ^3^Department of Agronomy, Iowa State University, AmesIA, USA; ^4^Department of Chemical and Biological Engineering, Iowa State University, AmesIA, USA; ^5^Department of Statistics, Iowa State University, AmesIA, USA

**Keywords:** *E. coli*, water quality, stream, surface property, particle

## Abstract

The importance of *E. coli* as an indicator organism in fresh water has led to numerous studies focusing on cell properties and transport behavior. However, previous studies have been unable to assess if differences in *E. coli* cell surface properties and genomic variation are associated with different environmental habitats. In this study, we investigated the variation in characteristics of *E. coli* obtained from stream water and stream bottom sediments. Cell properties were measured for 77 genomically different *E. coli* strains (44 strains isolated from sediments and 33 strains isolated from water) under common stream conditions in the Upper Midwestern United States: pH 8.0, ionic strength 10 mM and 22°C. Measured cell properties include hydrophobicity, zeta potential, net charge, total acidity, and extracellular polymeric substance (EPS) composition. Our results indicate that stream sediment *E. coli* had significantly greater hydrophobicity, greater EPS protein content and EPS sugar content, less negative net charge, and higher point of zero charge than stream water *E. coli*. A significant positive correlation was observed between hydrophobicity and EPS protein for stream sediment *E. coli* but not for stream water *E. coli*. Additionally, *E. coli* surviving in the same habitat tended to have significantly larger (GTG)_5_ genome similarity. After accounting for the intrinsic impact from the genome, environmental habitat was determined to be a factor influencing some cell surface properties, such as hydrophobicity. The diversity of cell properties and its resulting impact on particle interactions should be considered for environmental fate and transport modeling of aquatic indicator organisms such as *E. coli*.

## Introduction

Currently, pathogens are the leading cause of water quality impairments in rivers and streams in the United States, as often indicated by elevated levels of *E. coli* (USEPA, 2014). Therefore, improved understanding the variations of *E. coli* properties is needed for predicting fate and transport of the bacteria and to support the development of plans to reduce bacterial contamination of waters. Recent studies have indicated that there is high diversity of *E. coli* isolates in the environment (Lu et al., 2005; Bolster et al., 2009; Cook et al., 2011). This strain-level diversity has been described by differences in both genotype and phenotype, and therefore it likely impacts the fate and transport of *E. coli*. Moreover, bacterial survival and growth is a dynamic process affected by bacterial surface properties, such as extracellular polymeric substance (EPS), hydrophobicity, and net charge; both genomic and environmental factors regulate those properties (Goulter et al., 2009).

**FIGURE 1:**
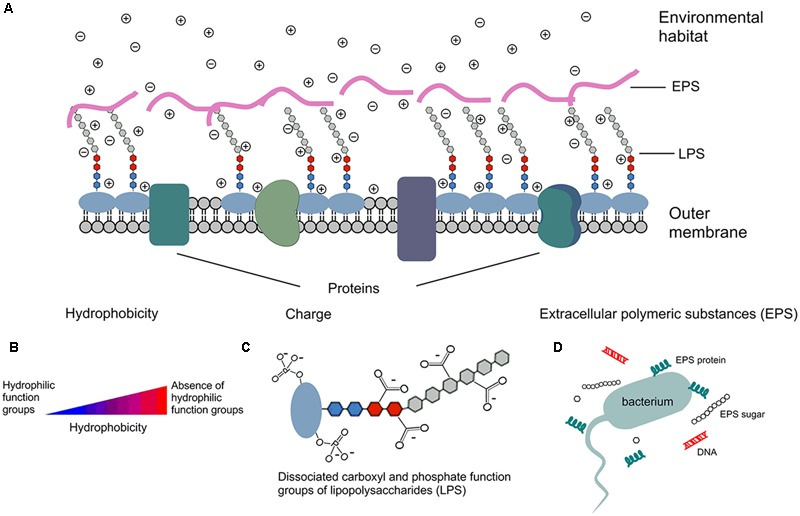
**Schematic depiction of *E. coli* surface properties.** ⊕ cation in solution; ⊖ anion in solution; - negative charge due to dissociation. **(A)** *E. coli* outer membrane has several components that contribute to cell surface properties, such as hydrophobicity, surface charge, zeta potential, and components of extracellular polymeric substance (EPS). **(B)** Cells that are absence of hydrophilic function groups are more hydrophobic than cells with hydrophilic function groups. **(C)** Surface is negatively charged due to the dissociation of carboxylic acid and phosphate functional groups; similar dissociation can also occur on the EPS and phospholipids of the membrane. Therefore, more cations than anions exist in solution, and that separation of charge is measured by the zeta potential. **(D)** EPS are mainly composed of sugars and proteins, but they may also include other macromolecules such as DNA.

Under typical stream pH in the Upper Midwestern United States (Dickson and Koohmaraie, 1989; Fein et al., 2005; Warnes et al., 2012), *E. coli* surfaces are negatively charged due to the dissociation of carboxyl and phosphate groups in the peptidoglycan and lipopolysaccharides of cell walls (Goulter et al., 2009; Warnes et al., 2012), as shown by **Figure 1**. While the magnitude of the surface charge of bacteria is highly environment-dependent (Fein et al., 2005), it can impact the bacterial state by repulsion of similarly charged particulates and by attraction of oppositely charged particulates (Dickson and Koohmaraie, 1989; Bolster et al., 2009). The hydrophobicity of a bacterial cell is determined by functional groups of both residues and structures on the surface of the cell, which can be either hydrophilic or hydrophobic (Vandermei et al., 1991). Hydrophobicity may change according to growth phase and growth condition, while the carbon content of the growth medium could positively impact hydrophobicity. Such impacts are partially due to the effects on lipid composition (Zikmanis et al., 2007). The presence of divalent cations, such as Ca^2+^ and Mg^2+^, could increase bacterial hydrophobicity (Khemakhem et al., 2005), since the cations principally attach to proteins on the bacterial surface and decrease their hydrophilicity (Jorand et al., 1998; Hoa et al., 2003; Wilen et al., 2003).

Extracellular polymeric substance are high-molecular-mass compounds secreted by microorganisms at the outer cell surface (Liao et al., 2015). They are mainly composed of polysaccharides and proteins, but they may also include other macromolecules such as DNA, lipids, and humic-like substances. EPS contribute to the overall heterogeneity of the bacterial surface (Walker et al., 2005; Zhao et al., 2014) and play an important role in cell aggregation, cell adhesion, and protection of cells from hostile environments (Dogsa et al., 2005; Vu et al., 2009; Bruckner et al., 2011). For example, the formation of biofilms in stream bottom sediments requires involvement of EPS (Sheng et al., 2010). The sugar/protein ratio of EPS has been positively correlated with the cell surface charge (Shin et al., 2001). Bolster et al. (2009) reported that EPS production mostly occurred in the late growth phase of bacteria. Moreover, EPS structure has been found to become more compact as environmental pH decreases (Dogsa et al., 2005).

Current water quality assessment techniques are based on environmental sampling, for which only the suspended populations of fecal indicator bacteria are collected (Bai and Lung, 2005; Pandey and Soupir, 2013); this procedure does not assess microbial contamination of stream bottom sediments. However, previous research has indicated that after entering surface waters, microorganisms often partition into the planktonic state or they attach to suspended soil and organic particles (Jeng et al., 2005; Hipsey et al., 2006; Pachepsky et al., 2008; Liang et al., 2014). The populations of bacteria surviving in bottom sediments are protected from ultraviolet radiation (Bitton et al., 1972; Schillinger and Gannon, 1985), resulting in an extended survival time. When stream bottom sediments are disturbed during changes in flow, there is increasing likelihood of resuspension back into the water column (Song et al., 1994; Davies and Bavor, 2000; Jamieson et al., 2005). Therefore, improved understanding of the properties of sediment-associated *E. coli* is critically important for understanding bacterial fate in the environment.

An assessment of the variation of *E. coli* cell properties in different environments (stream bottom sediments versus the overlying water column) is needed to better understand environmental fate and transport. In this study, we divided the potential impacts on bacterial surface properties into two parts: genomic impact (intrinsic) and environmental impact (extrinsic). The overall goal was to determine if differences in *E. coli* cell surface properties were due primarily to extrinsic or intrinsic properties or to an interaction of the two. We assessed 77 genomically distinct *E. coli* strains with respect to hydrophobicity, zeta potential, net charge, total acidity, and EPS composition (protein and sugar). The objectives of our study were: (1) to compare bacterial properties between *E. coli* isolated from two environmental habitats, stream sediments, and stream water; (2) to determine the correlations among bacterial surface properties within each environmental habitat and compare the correlations obtained from different environmental habitat; (3) to explore the relationship between genomic similarity and environmental habitat; and (4) to investigate bacterial surface properties as a function of environmental impact (extrinsic), regardless of genomic similarity (intrinsic).

## Materials and Methods

To investigate the potential impacts from intrinsic genomic and extrinsic environmental aspects, *E. coli* strains collected from two environmental habitats were studied. For each *E. coli* strain, several bacterial surface properties were measured: hydrophobicity, zeta potential, net charge, total acidity, and EPS composition by extraction and colorimetric techniques. Genome similarities were also analyzed for each pair of *E. coli* strains.

### *E. coli* Sampling and Analysis

Stream sediment and water were collected six times from two locations along Squaw Creek in Ames, IA, USA, in 2012 and 2013: Cameron School Road (latitude 42.0707, longitude -93.6728), and Brookside Park (latitude 42.0290, longitude -93.6288). Water samples were collected by lowering a horizontal polycarbonate water bottle sampler (2.2 L, Forestry Suppliers Inc., Jackson, MS, USA) from a bridge into the center of the creek at both of the locations. Sediment samples were collected from the top 2–3 cm of the streambed using a shallow water bottom dredge sampler (15 cm × 15 cm opening, Forestry Suppliers Inc., Jackson, MS, USA) at the same location as the water samples were collected. Immediately after collection, samples were placed on ice. The sediment-associated *E. coli* were detached by stirring a mixture of sediment and deionized water (ratio 1:1) for 15 min at approximately 200 rpm using a magnetic stir bar under room temperature (Pandey et al., 2012). One milliliter of the resulting sediment solution was filtered through a 0.45-μm cellulose filter paper (EMD Millipore; Pittsburg, PA, USA). *E. coli* strains were incubated on the filter paper using modified mTEC agar plates (USEPA, 2002). A single colony was selected from each agar plate and the plate-streaking method was applied to ensure that the selected colony was formed by only one *E. coli* strain. Two hundred strains were isolated from the stream sediment. Each 100-mL water sample was filtered through a 0.45-μm filter paper, and another 200 strains were obtained from the water samples. After isolation, frozen stocks were made according to standard technique. The strains were inoculated in Luria–Bertani liquid media (BD Biosciences; San Jose, CA, USA), grown to the stationary phase, and stored at -80°C in 15% glycerol.

### Computer-Assisted Rep-PCR DNA Fingerprint Analysis

Rep-PCR was performed (Rademaker and de Bruijn, 1997) with (GTG)_5_ (5′-GTGGTGGTGGTGGTG-3′) as primer (Mohapatra and Mazumder, 2008; Mohapatra et al., 2008; Ma et al., 2011). The PCR reaction contained 12.5 μL PCR-master-mix (2X, Qiagen), 10 μL primer (50 pmol), and 2.5 μL water. A small fraction of a fresh *E. coli* colony was transferred to the PCR mixture as the template by using a 1-μL loop. PCR was conducted in a C1000 Thermal Cycler (Bio-Rad, Hercules, CA, USA). The thermo cycler program was set for an initial denaturation (95°C for 2 min), 32 cycles of denaturation (94°C for 3 s and 92°C for 30 s), annealing (40°C for 1 min), extension (65°C for 8 min), and a final extension (65 °C for 8 min). Then 10 μL of resulting PCR products and 2 μL of 6X loading dye mixture (Life Technology, Grand Island, NY, USA) were loaded onto 1.5% agarose gel, and the 1 Kb Plus DNA ladder (Life Technology, Grand Island, NY, USA) was loaded into every tenth well and was used as an external control for normalization. Electrophoresis was applied at 4°C and 80 V for 13.5 h, and the sample was stained for 20 min in TAE solution containing 0.5 μg mL^-1^ ethidium bromide. Gel pictures were captured with a Molecular Imager ChemiDoc (Bio-Rad, Hercules, CA, USA).

The resulting gel image files were imported into Bionumerics (version 7.1, Applied Maths, Kortrijk, Belgium) for normalization, band identification, and cluster analysis. Bands of more than 5000 bp and less than 300 bp were eliminated from analysis to avoid false clustering. Similarity coefficients for each strain pair were generated by Pearson’s correlation method with a band matching tolerance of 0.5%, and an optimization value of 0.5% (BioNumerics, 2013). Unweighted pair groups with mathematical averages (UPGMA) was used for clustering and generating the dendrogram. Strains with similarity less than 90% were considered genomically different. *E. coli*- (GTG)_5_ genomic similarity matrix was also obtained from Bionumerics.

Genomically different *E. coli* strains were assigned to one of seven phylotypes (A, B1, B2, A/C, D/E, E, or F) using the revised Clermont method (Clermont et al., 2013). Strains were inoculated in M9 broth (minimal media) with 0.4% (w/w) glucose at 37°C and incubated to early stationary phase (OD600 = 1.0–1.5). To harvest the cells, *E. coli* were centrifuged for 15 min at 4,000 rpm/1878 × *g* (Centrifuge 5430R with Rotor F-35-6-30, Eppendorf, Hauppauge, NY, USA) at 4°C. The supernatant was discarded, and the cell pellet was used for property analysis.

### Cell Properties

The microbial adhesion to hydrocarbon (MATH) method was employed to estimate the hydrophobicity of the *E. coli* strains (Rosenberg et al., 1980; Pembrey et al., 1999). Briefly, the cell pellet was resuspended in 4 mL of deionized water and OD546 of the cell suspension (initial OD546) was measured by spectrophotometer (HACH, Loveland, CO, USA). Then the cell suspension was transferred to individual glass test tubes (1.7 cm in diameter, 15 cm in length), each of which contained 1 mL of dodecane (99%, Fisher Scientific, Fair Lawn, NJ, USA). The tubes were vortexed (Fisher Scientific, Fair Lawn, NJ, USA) at full speed for 2 min and then left vertically at room temperature for 15 min for phase separation. The OD546 of the aqueous phase was determined and hydrophobic partitioning of the bacterial suspension was calculated by using this equation from Pembrey et al. (1999): hydrophobic partitioning = (initial OD546 - OD546 of aqueous phase)/initial OD546. The analysis was performed in triplicate.

Zeta potential measurements were performed at room temperature using a Zetasizer Nano-ZS. To mimic typical stream environments of the Upper Midwestern United States, a solution of CaCO_3_ was prepared by diluting saturated CaCO_3_ solution to pH 8.0 and an ionic strength of 10 mmol L^-1^. The *E. coli* cell pellet was washed twice with CaCO_3_ solution then suspended in CaCO_3_ solution to OD600 = 0.1. The resulting suspension was poured into a disposable capillary cell (DTS1070). The average and standard deviation of 12 runs were recorded.

Potentiometric titration of *E. coli* cells was conducted to measure the acidity of the bacterial surface. The harvested cell pellet was suspended in CaCO_3_ solution (pH = 8.0, ionic strength of 10 mmol L^-1^). The concentration of *E. coli* cells in the suspension was then determined by cellometer (Auto M10, Nexcelom Bioscience LLC, Lawrence, MA, USA). Then the solution pH was adjusted to 4.0 by addition of 0.01 mol L^-1^ HCl. Next, the *E. coli* suspension was purged with nitrogen gas for 1 h to remove dissolved carbon dioxide (Walker et al., 2005), and then it was titrated with NaOH (0.01 mol L^-1^) from pH 4.0 to 10.0 using a titrator (Multitasking titration system, Lab synergy, Goshen, NY, USA). A blank titration with CaCO_3_ solution without *E. coli* was run separately. The number of moles of deprotonated sites was calculated as described by Fein et al. (2005):

$${\left[NC\right]}_{\text{Net}\;\text{charge}\;\text{meq}\;\text{per}\;{\text{10}}^{\text{8}}\;\text{cells}}=\left[\frac{\begin{array}{c} {\left(C_{\text{A}}-C_{\text{B}}-[H^{+}]-[OH^{-}]\right)}_{\text{sample}}\\ -\;{\left(C_{\text{A}}-C_{\text{B}}-[H^{+}]+[OH^{-}]\right)}_{\text{blank}} \end{array}}{N_{\text{bact}}}\right]\times 10^{-3}$$

where N_bact_ is the total number of cells per mL of solution obtained by the cellometer; C_A_ and C_B_ are the concentrations (in mmol L^-1^) of acid and base (including initial amounts of acid or base added to the suspension prior to the titration); [H^+^] and [OH^-^] are the concentrations of H^+^ and OH^-^, calculated from themeasured pH. The net charge was determined as the difference of charge between the *E. coli* suspension sample and the blank. The total acidity was obtained by subtracting the net charge at pH 10.0 from the net charge at pH 4.0. The surface charge at pH 8.0 and the point of zero charge (PZC) were also points of interest. The sample analyses were performed in duplicate while the blank solutions were titrated in triplicate and averaged. **Supplementary Figure A1** shows an example of the potentiometric titration curve to demonstrate the useful information which can be obtained from this measurement.

The EPS, specifically the total protein and the polysaccharide content, was determined by an extraction method (Chang, 2005). Briefly, *E. coli* cells were incubated on a 0.45-μm filter membrane on multiple mTEC agars overnight at 37°C to obtain the total amount of *E. coli* cell within the range of 3 × 10^10^ to 6 × 10^10^ cells, and then the membrane was placed in 30 mL of 0.85% (w/v) NaCl solution. The *E. coli* concentration was measured by cellometer. After centrifugation at 16,300 × *g* for 30 min at 4°C, the supernatant was filtered through a 0.45-μm filter. The filtrate was then added to 90 mL of ice-cold 100% ethanol and stored at -20°C for 24 h. Finally, the EPS pellet was harvested by centrifugation at 16,300 × *g* for 30 min at 4°C and air-dried in a fume hood. The analysis of EPS protein was conducted using the Lowry method (Lowry et al., 1951), which is a spectrometric method based on measurement at a wavelength of 500 nm using bovine serum albumin (Sigma-Aldrich, St. Louis, MO, USA) as the standard. The EPS sugar was analyzed by the phenol-sulfuric acid method, which is based on measurement at a wavelength of 488 nm using xanthan gum as the standard (Dubois et al., 1956; Im et al., 2010).

### Data Analyses

Statistical analysis of data was performed using R project software (version 3.1.3, Institute from Statistics and Mathematics, Vienna University of Economics and Business, Vienna, Austria). The non-parametric Wilcoxon signed-rank test was used to determine if any of the properties varied between sediment *E. coli* strains and water *E. coli* strains. To investigate the correlation between any two *E. coli* properties, the Kendall-tau correlation method and the LOESS-smoothing method were applied. The Mantel test was conducted to determine the correlation between *E. coli*- (GTG)_5_ genomic similarity and environmental habitat (stream bottom sediments or overlying water). Moreover, phylogenetic generalized least squares was employed to explore the impact of environmental habitat on bacterial properties, by excluding the potential impact from genomic similarity. Phylogenetic methods are used in the analysis of interspecies data because species are non-independent for statistical analysis (Revell, 2010). When applying the phylogenetic generalized least squares analysis (R package ‘pGLS’), one bacterial property was considered as the response (***Y***), environmental habitat was considered as the binary predictor (***X***), and similarity matrix was considered as the variance-covariance matrix. The method uses a variance-covariance matrix to weight the predictors (Mao, 2015).

## Results and Discussion

### *E. coli* Strain Selection, Dendrogram, and Phylo-Type

**FIGURE 2:**
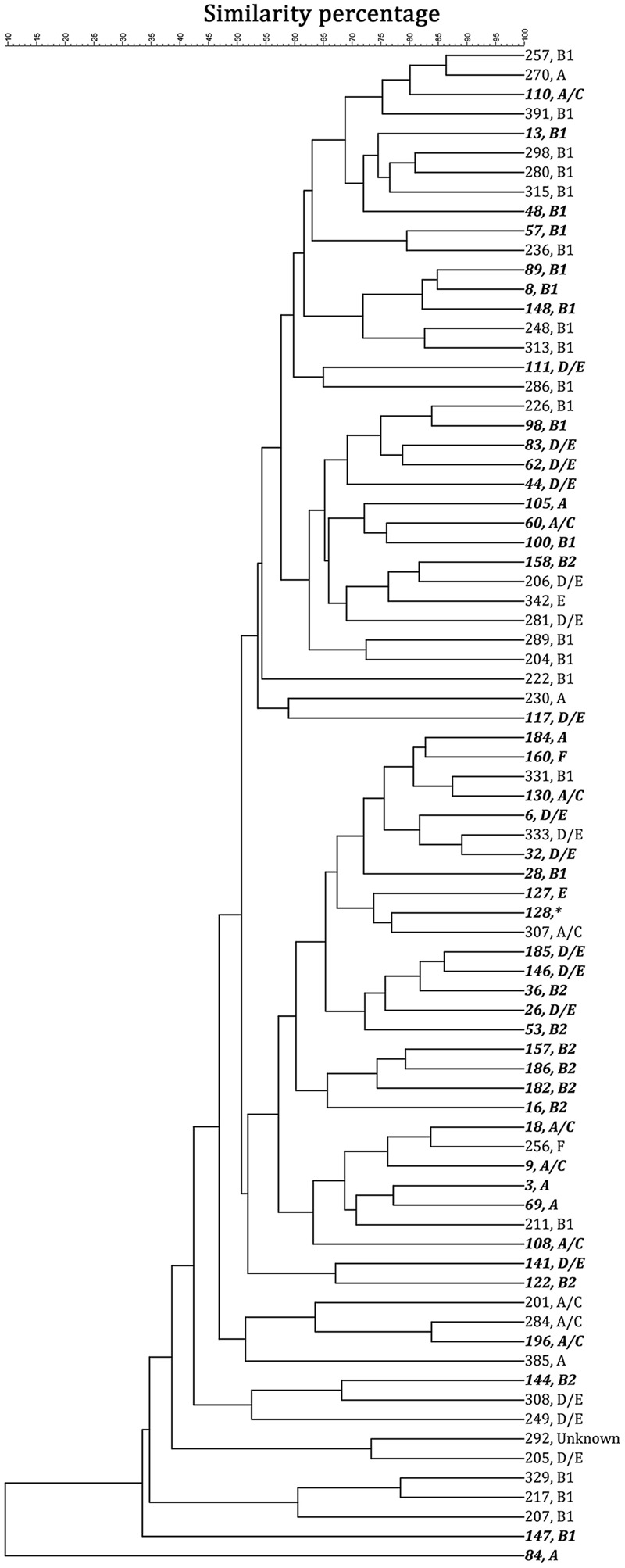
**The 78 strains selected for characterization showed less than 90% genomic similarity, based on a unweighted pair groups with mathematical averages (UPGMA) cluster analysis.** Strains 1–200 (italic and bold) were collected from stream sediment; while 201–400 were collected from stream water. The strain number is followed by the phylotype analysis. ^∗^Strain 128 could not be recovered for phylotype analysis.

By computer-assisted rep-PCR DNA fingerprint analysis, 45 sediment strains (22.5% of 200 strains) and 33 water strains (16.5% of 200 strains) were considered genomically distinct on the basis of the 90% similarity criterion. Phylo-type analysis was conducted to query the origin of the isolates and to put the work into the context of other recently published environmental studies. **Figure 2** shows the dendrogram of these 78 selected strains based on the genome similarity score from the rep-PCR fingerprint as well as the phylo-type, while **Supplementary Figure A2** shows the dendrogram of the electrophoresis image. There was no obvious cluster pattern for *E. coli*-(GTG)_5_ genomic profiles of strains from the same environmental habitat (stream water or sediment). Phylo-typing revealed that sediment isolates had more diverse phylo-type (25% classified as D/E, 23% as B1, 20% as B2, 16% as A/C, 11% as A, 2% as E, and 2% as F) when compared to water isolates in which 56% of isolates were classified as B1 (additionally, 19% classified as D/E, 9% classified as A, 9% classified as A/C, 3% as E, and 3% as F). Previously *E. coli* from phylogenetic groups A and B1 were associated with livestock (Cortes et al., 2010; Schulz et al., 2016) while B2 and D strains are more likely to be associated with extraintestinal infections. Note that one sediment strain (strain No. 122) had insufficient growth in M9 broth to proceed, so further analyses were based on 44 sediment strains and 33 water strains. This analysis demonstrates that each of the isolates characterized here was genomically distinct from the others, and variation in phylo-types existed.

### *E. coli* Property Comparison between Two Environmental Habitats

**FIGURE 3:**
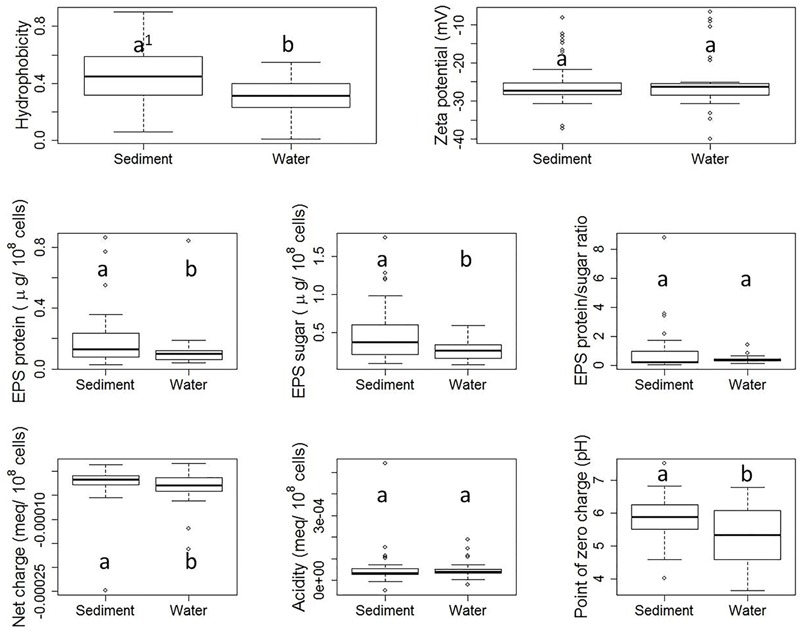
**Boxplots of property results from stream sediment *E. coli* and stream water *E. coli*.** Each plot shows five numerical values: the smallest observation ( [Q1 = Q2 - 1.5(Q4 - Q1) ], the low end of the whisker), 25% quartile (Q2, low boundary of box), median (Q3, the band near the middle of box), 75% quartile (Q4, high boundary of box), and largest observation ( [Q5 = Q4 + 1.5(Q4 - Q1) ], the high end of the whisker). Outliers, if any, are indicated by dots. Within each subplot, values with the same letter are not different at the significant level. The difference is determined by a Wilcoxon test with significance level set at α = 0.05.

Although each *E. coli* strain was subjected to the same storage and growth conditions, diversities in properties of *E. coli* derived from stream water and sediment were observed, as shown by **Figure 3**. Hydrophobicity, as measured by the MATH assay, ranged from 0.01 to 0.90. Zeta potential ranged from -6.76 to -39.87 mV. Total EPS protein content ranged from 0.30 to 0.86 μg/10^8^ cells, while total sugar content of EPS ranged from 0.80 to 1.74 μg/10^8^ cells. The EPS protein/sugar ratio ranged from 0.07 to 8.78. Net charge at pH 8.0 varied from -2.48 × 10^-4^ to 1.60 × 10^-5^ meq/10^8^ cells. Variation was also observed in the total acidity and PZC. While previous studies have reported surface property ranges for *E. coli* isolates, here we have measured all of these properties for this large number of distinct isolates. This framework could be applied to other organisms.

**Figure 3** shows the boxplots of property results analyzed for sediment *E. coli* strains and water *E. coli* considered separately. Statistically significant differences in cell properties were observed between stream sediment *E. coli* and water *E. coli* in many cases. For example, according to the Wilcoxon test, sediment *E. coli* had a significantly greater hydrophobicity than water *E. coli* (*p*-value = 0.005). Previously, Stenstrom (1989) and Zita and Hermansson (1997), reported that higher hydrophobicity coincided with greater adhesion to mineral particles in water and sludge flocs in sludge liquor from wastewater treatment plants. In addition to hydrophobicity, sediment *E. coli* also had greater EPS protein content (*p*-value = 0.006) and sugar content (*p*-value = 0.036), less negative net charge (*p*-value = 0.026), and higher PZC (*p*-value = 0.009) than stream water *E. coli*.

The zeta potential of suspended *E. coli* cells reflects the electrokinetic potential of the cell surfaces. Colloid stability (i.e., the likelihood that cells will not coagulate with one another) will increase as the absolute value of the zeta potential increases. Previous studies have indicated that zeta potential ranged from -4.9 to -29 mV for 280 different *E. coli* strains (Morrow et al., 2005) or ranged from -3.5 to -49 mV for 12 different *E. coli* strains (Bolster et al., 2009), which are similar with the range of our results. In our study, there was no significant difference between zeta potentials measured for sediment *E. coli* strains and water *E. coli* strains. Similarly, previous research has found no clear correlation between the zeta potential of bacterial cells and their adhesion to a negatively charged polystyrene surface (van Loosdrecht et al., 1987) or quartz particles (Bolster et al., 2009).

Our results also indicated that stream sediment *E. coli* strains had significantly higher EPS protein and sugar content than stream water strains, which is consistent with the fact that cell adhesion and biofilm formation require EPS (Dogsa et al., 2005; Vu et al., 2009). The EPS protein/sugar ratio can be dramatically impacted by environmental pH (Dogsa et al., 2005), culturing time (Shin et al., 2001), culturing medium, and extraction method (Nielsen Per and Jahn, 1999; Sheng et al., 2010). However, while the absolute values of EPS protein and sugar contents differed between the two habitats, no significant difference in the EPS protein/sugar ratio was observed in our study.

Moreover, the net charge at pH 8.0 of stream sediment *E. coli* was significantly less negative than the net charge of water *E. coli*. *E. coli* cells in both water and sediment carry an overall negative surface charge at pH 8.0. Those strains carrying more negative net charge need to overcome a greater barrier from electrostatic force when attaching to negatively charged sediment surfaces, as demonstrated in previous studies (Dickson and Koohmaraie, 1989; Bolster et al., 2009). The cell surfaces of sediment *E. coli* also had a lower PZC.

### *E. coli* Property Correlations

**FIGURE 4:**
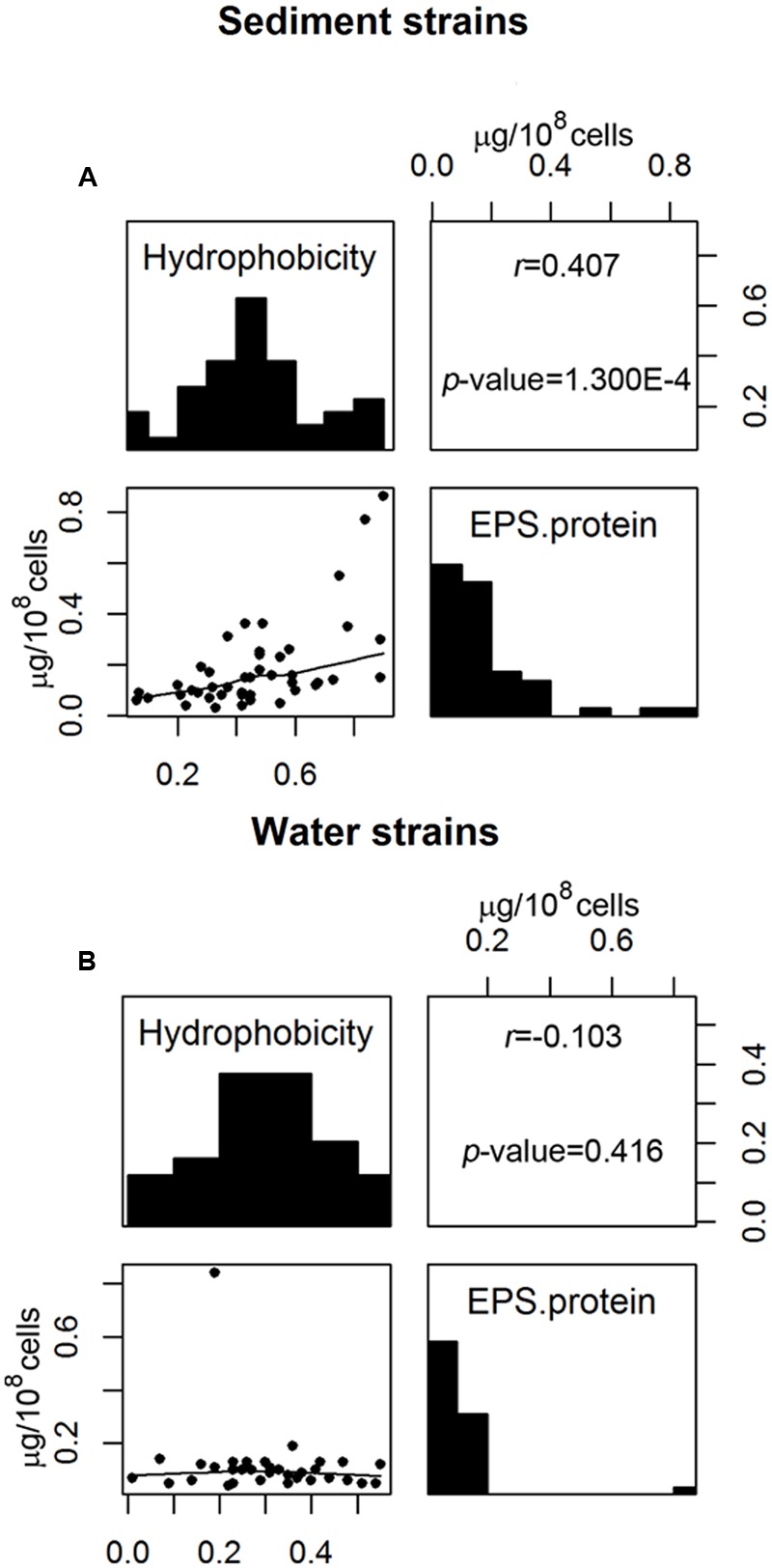
**Correlation between *E. coli* hydrophobicity and EPS protein content for **(A)** sediment *E. coli* strains and **(B)** water *E. coli* strains.** The upper panels show the correlation coefficient *r* with associated *p*-value analyzed by Kendall-tau method; the diagonal panels show histograms of each property; the lower panels are scatterplots with the smoothing curves using the Lowess method.

Correlation analyses were used to determine whether any two measured cell properties were dependent. Pair-wise correlations between the different cell properties measured in this study were generally low and not statistically significant when analyzed for all 77 *E. coli* strains (**Table 1**). Of the statistically significant correlations observed in this study, some were between properties which had shared parameters or measurements so the correlations were artificially inflated. Such correlations include: the EPS protein/sugar ratio with EPS protein and EPS sugar, net charge with acidity, and net charge with PZC. On the other hand, the strong positive correlation between hydrophobicity and EPS protein content [*r* (correlation coefficient) = 0.283; *p*-value = 3.914 × 10^-4^] is novel and useful. Interestingly, the correlation between hydrophobicity and the EPS protein was not the same for stream sediment *E. coli* and water *E. coli*. Using scatterplots with smoothing curves, histograms of each property, and the results from the Kendall-tau correlation method, **Figure 4** shows the correlations between *E. coli* hydrophobicity and EPS protein content for stream sediment *E. coli* and water *E. coli*, respectively. For sediment *E. coli*, there was significant positive correlation (*r* = 0.407, *p*-value = 1.274 × 10^-4^) between hydrophobicity and the EPS protein; while for water *E. coli*, no significant correlation was observed (*r* = -0.103 with *p*-value = 0.416). Previous research has found that hydrophobic components of EPS are mainly comprised of proteins (Jorand et al., 1998; Gerbersdorf et al., 2008). Perhaps the identity or abundance of those EPS proteins lead to the partitioning of these strains into the sediment where they can be protected from environmental stresses such as ultraviolet radiation. Additional studies to characterize EPS proteins associated with the two groups of *E. coli* would be helpful to determine the mechanisms behind these observations.

**Table 1:** Correlation coefficient matrix for cell properties for all *E. coli* strains (*n* = 77) obtained from Kendall-tau correlation method.

	Hydrophobicity	Zeta potential	EPS protein	EPS sugar	Ratio (EPS protein/sugar)	Net charge at pH 8.0	Acidity
Zeta potential	-0.047						
Extracellular polymeric substance (EPS) protein	***0.283***	-0.179					
EPS sugar	-0.060	-0.177	0.095				
Ratio (EPS protein/sugar)	0.203	-0.016	***0.403***	**-*0.522***			
Net charge at pH 8.0	0.131	0.078	0.035	-0.141	0.066		
Acidity	0.016	-0.085	0.148	0.064	0.078	**-*0.540***	
Point of zero charge	0.237	0.075	0.149	-0.121	0.136	***0.403***	-0.170

*Statistically significant (p < 0.001) are in italic and bold.*

By definition, surface charge and zeta potential are related. However, our results indicate only a very weak correlation between net charge and zeta potential with *r* = 0.008 with *p*-value = 0.316. Thus our results may temper the conclusions of some previous research in which zeta potential has been used to estimate surface charge (Alves et al., 2010; Zhang et al., 2012).

### Correlation between Genomic Similarity with Environmental Habitat

To explore possible relationships between *E. coli*-(GTG)_5_ genomic similarity and environmental habitat (stream sediment or water), the Mantel test was applied. The results showed a significantly positive correlation between genomic similarity and environmental habitat (*r* = 0.063; *p*-value = 0.002). This finding indicates that genomic similarity was larger within habitat, as compared to between habitat types. Thus, genomic factors may ultimately influence whether certain strains remain in the water or sediment.

### *E. coli* Property Comparison Integrating Phylogenetic Correlation

Phylogenetic generalized least square method was employed to explore the impact of environmental habitat (extrinsic) on bacterial properties, while excluding potential impacts from genomic similarity (intrinsic). The results indicate that some bacterial properties were significantly regulated by environmental habitat: e.g., *E. coli* strains occurring in stream sediments were more likely to have higher hydrophobicity (*p*-value = 2.935 × 10^-7^), EPS sugar content (*p*-value = 3.953 × 10^-3^), net charge (*p*-value = 3.414 × 10^-4^), and PZC (*p*-value = 6.165 × 10^-8^), but lower acidity (*p*-value = 0.016) when compared to *E. coli* strains suspended in the water column.

Previous studies have been unable to determine if differences in environmental *E. coli* cell surface properties and genomic variation residing in different environmental habitats (stream bottom sediments versus overlying water) are due primarily to environmental habitat (extrinsic), genomic similarity (intrinsic), or an interaction of these two. Below is a summary of our major conclusions:

• Statistically significant differences in cell properties were observed between stream sediment *E. coli* and water *E. coli*; most notably, sediment *E. coli* had significantly greater hydrophobicity, EPS protein content, and EPS sugar content; less negative net charge; and higher PZC when compared to water *E. coli*.
• Hydrophobicity and EPS protein were positively correlated for stream sediment *E. coli* but not for water *E. coli*.
• Genomic similarity was greater within environmental habitat, as compared to between habitat types.
• When the impacts of genomic similarity were accounted for, the impact of environmental habitat on hydrophobicity, EPS sugar, net charge, PZC, and acidity was significant among the strains, indicating that habitat was a regulating factor for expression of these properties.

## Author Contributions

CL, XL, and MS collected strains. XL created **Figure 1**, performed the experiments for and analyzed the data presented in **Figures 2–5**, wrote the manuscript draft, and finalized the manuscript. CL performed rep-PCR. MT, MS, and LJ contributed to experimental design, data analysis, and manuscript writing. PD contributed to statistical analysis.

## Conflict of Interest Statement

The authors declare that the research was conducted in the absence of any commercial or financial relationships that could be construed as a potential conflict of interest.

The reviewer GDO and handling Editor declared their shared institutional affiliation and the handling Editor states that the process nevertheless met the standards of a fair and objective review.

## Supplementary Material

FIGURE A1 **Potentiometric titration curve example from strain number 53.** Three pieces of information can be obtained from the curve: (1) Point of zero charge of *E. coli* strain = pH 5.8; (2) Net charge at pH 8.0 carried by *E. coli* = -7.5 × 10^-6^ meq/10^8^ cells; (3) Total acidity of *E. coli* strain = 2.8 × 10^-5^ meq/10^8^ cells.

FIGURE A2 **The dendrogram shows the percent similarity of rep-PCR fingerprint banding patterns for 78 strains with similarity smaller than 90%, based on UPGMA cluster analysis.** Strains 1–200 (italic and bold) were collected from stream sediment, while 201–400 were collected from stream water.
