# Supplementary figures and images for: E. coli Surface Properties Differ between Stream Water and Sediment Environments
Source: Front Microbiol. 2016 Nov 1;7:1732. doi: 10.3389/fmicb.2016.01732 (PMC5088573; doi:10.3389/fmicb.2016.01732)

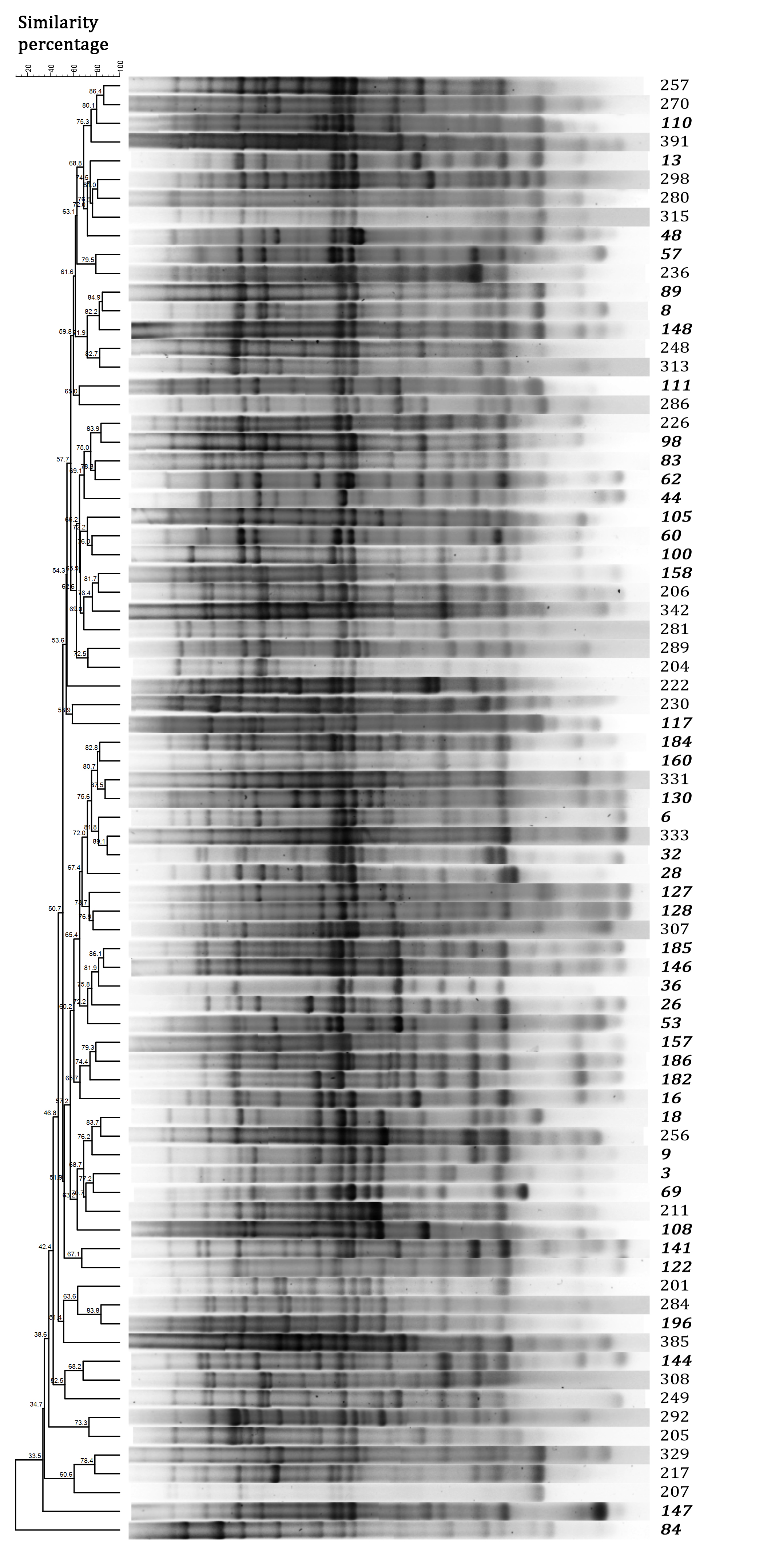

Supplement: FIGURE A2 — The dendrogram shows the percent similarity of rep-PCR fingerprint banding patterns for 78 strains with similarity smaller than 90%, based on UPGMA cluster analysis. Strains 1–200 (italic and bold) were collected from stream sediment, while 201–400 were collected from stream water. [file Image_2.JPEG]
